# Supplementary material for: Elevated ozone disrupts mating boundaries in drosophilid flies
Source: Nat Commun. 2024 Apr 11;15:2872. doi: 10.1038/s41467-024-47117-7 (PMC11009341; doi:10.1038/s41467-024-47117-7)
Supplement: Supplementary file 1 — Supplementary Information [file 41467_2024_47117_MOESM1_ESM.pdf]

Title: Elevated ozone disrupts mating boundaries in drosophilid flies

Nan-Ji Jiang[1,2], Xinqi Dong[1], Daniel Veit[3], Bill S. Hansson[1,2,+], Markus Knaden[1,2,+,*]

[+]authors contributed equally
*corresponding author: mknaden@ice.mpg.de

Affiliations:

[1]Department of Evolutionary Neuroethology, Max Planck Institute for Chemical Ecology, Hans-Knöll-Straße 8, D-07745 Jena, Germany

[2]Next Generation Insect Chemical Ecology, Max Planck Centre, Max Planck Institute for Chemical Ecology, Hans-Knöll-Straße 8, D-07745 Jena, Germany

[3]Max Planck Institute for Chemical Ecology, Hans-Knöll Straße 8, D-07745 Jena, Germany

This PDF file includes:

Supplementary discussion, Supplementary Figures 1-6, and legends.

Supplementary Discussion

Reproductive isolation i.e. the lack of gene flow between populations is regarded as an important driver of speciation (reviewed by Mallet, 2006)[1]. Such reproductive isolation often is a result of geographic isolation of so-called allopatric populations that via different selective pressures or genetic drift become more and more dissimilar and finally speciate. In addition, few examples of sympatric speciation (i.e. the evolution of a new species in close proximity of its ancestral species) have been identified in e.g. African cichlids[2-4] or the apple maggot fly[5-7]. Finally, some species seem to be the result of hybrid speciation[8], where the hybridization between closely related species finally results in the evolution of a new species. The most prominent insect example is the species-rich genus of *Heliconius* butterflies[9], where hybridization of two closely related species can result in a fertile hybrid that by its wing pattern and behavior is reproductively isolated from the two donor species[10]. Similarly, there is one reported case of hybrid speciation for *Drosophila*, where hybrids of *D. ananassae* and *D. parapallidosa* obviously evolved into the new species *D. cf. parapallidosa*[11].

Our manuscript deals with four species of the *Drosophila melanogaster* complex, because both their pheromone blends and their sexual behavior are well established. *D. sechellia*, and *D. mauritiana* most probably have evolved from a large mainland population of a shared ancestor with *D. simulans* through allopatric speciation based on two island colonization events[12]. *D. simulans*, like *D. melanogaster* nowadays is globally distributed and also occurs on the Mauritius and the Seychelles, i.e. the islands originally inhabited by *D. mauritiana* and *D. sechellia*. It has been shown that in *Drosophila* flies during speciation usually first prezygotic isolation (i.e. via courtship and mating boundaries) and afterward postzygotic isolation (via hybrid sterility and inviability) are established[13,14]. *D. simulans*, *D. sechellia*, and *D. mauritiana* belong to the *simulans* species complex and have established prezygotic isolation based on e.g. species-specific pheromonal blends[15-18] and courtship songs[19-21]. Their post-zygotic isolation, however, is incomplete, as only male hybrids are sterile, while female hybrids are fertile. On both islands, gene flow via hybridization events between *D. simulans* and its close relatives has been reported (with *D. sechellia*[22]; with *D. mauritiana*[23]), suggesting that presynaptic isolation between these

species is not absolute. Our data reveal, that oxidant pollutants like ozone have the potential to corrupt prezygotic isolation and, hence, make hybridization events more likely. As at least some of the resulting hybrids seem to be competitive regarding mate choice (Fig. 3) and reproduction (Fig. S6), such hybridization events potentially could result in ongoing gene flow between sympatric species. As for these species several genetic incompatibilities have been reported[24-28], it, however, is questionable, whether ongoing gene flow in this species complex has the potential to finally result in hybrid speciation. In addition, within the *Drosophila* genus, however, many more sympatric species pairs exist that can hybridize[29] and whose species boundaries therefore might also become affected by increased levels of ozone.

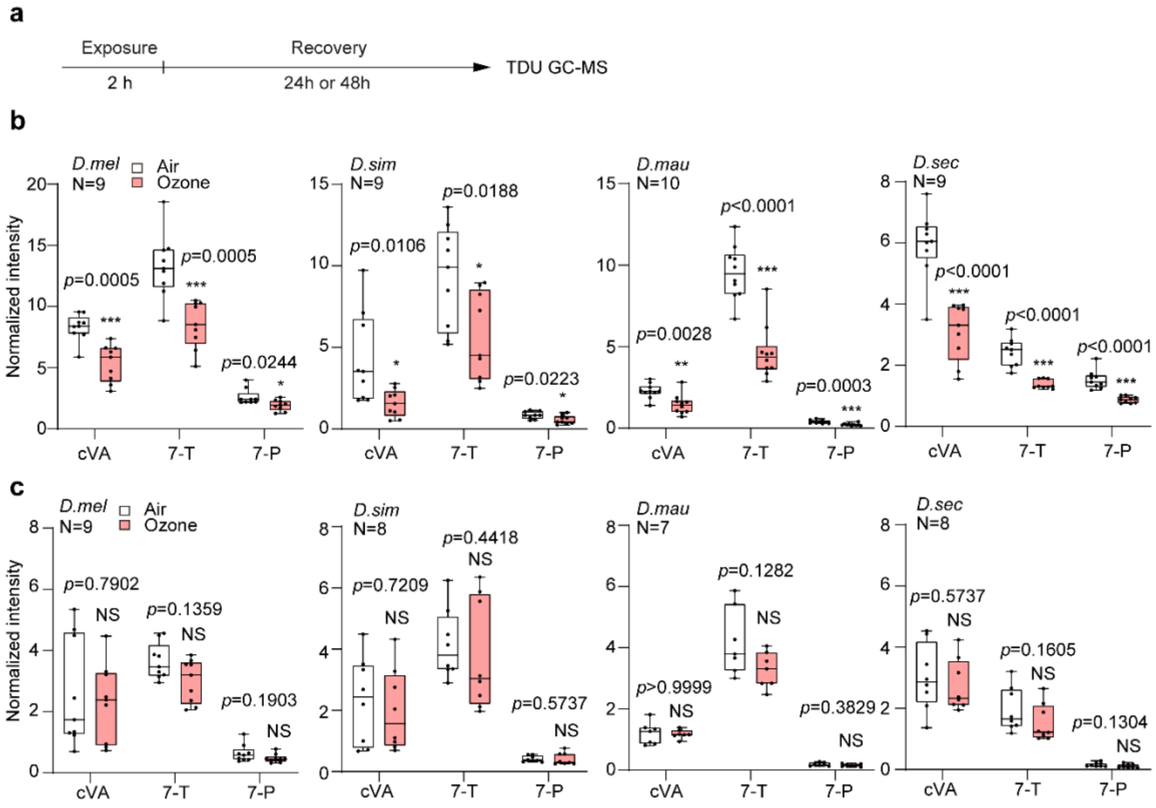

Figure S1. Quantitative analysis of cVA and pheromonal CHCs after ozone exposure recovery in four *Drosophila* species. a, Time line of experiment. Ozonated and control flies are exposed for two hours to 100 ppb ozone and ambient air, respectively. After that flies were placed into food vials and we let them recover for 24h or 48h. **b**, Quantitative analysis after 24h recovery. **c**, Quantitative analysis after 48h recovery. The box plots present median values and quartiles, whiskers the minimum and maximum values, and dots the individual data points. Two-sides *Unpaired t*-test. **p*<0.05; ***p*<0.01; ****p*<0.001; NS, no significant difference. Because of the GC-MS components e.g. ion source, column, and the concentration of internal standards are varying, hence we only compare our results from the same test sequence to minimize the variations.

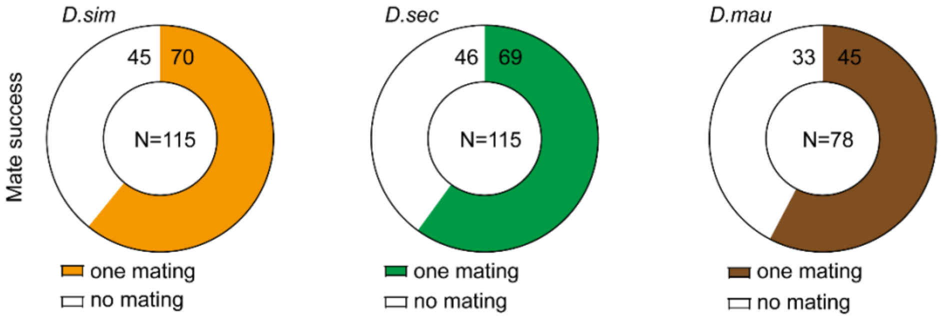

Figure S2. Mating frequency during 6 hours when a female can choose between two conspecific males. The numbers in the donut plots indicate the experiments that resulted in single mating (colored) or no mating (white). We never observed that a female mated twice during these 6 hours. *D. sim*: *D. simulans*; *D. sec*: *D. sechellia*; *D. mau*: *D. mauritiana*.

a

An overview of hybridization between *D.mel*, *D.sim*, *D.mau*, and *D.sec.*

♀/♂	*D.mel*	*D.sim*	*D.sec*	*D.mau*
D.mel	--	*D.mel-sim* no male	*D.mel-sec* no male	*D.mel-mau* no male
D.sim	*D.sim-mel* only male	--	*D.sim-sec* both sexes	*D.sim-mau* both sexes
D.sec	*D.sec-mel* only male	No hybrid<br>No hybrid	--	Not tested
D.mau	*D.mau-mel* only male	No hybrid obtained<br>*D.mau-sim* both sexes	Not tested	--

--: no hybridization.

Black: obtained hybrids in this study.

Grey: reported hybrids in previous studies.

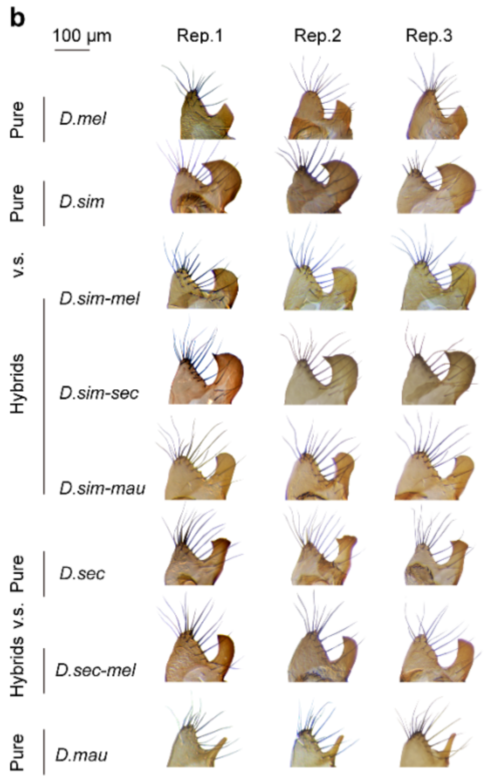

Figure S3. A hybridization overview between four *Drosophila* species and male posterior lobe morphology of *Drosophila* purebred species and hybrids. **a**, Hybridizations between four *Drosophila* species. Black letters, hybrids obtained in this study; gray letters, hybrids reported by previous references. **b**, Morphology of male posterior lobes. *D. mel*: *D. melanogaster*; *D. sim*: *D. simulans*; *D. sec*: *D. sechellia*; *D. mau*: *D. mauritiana*. All hybrids are F_1 and named as F_0 female × F_0 male, e.g. *D. sim-mel* is a hybrid offspring of a female *D. sim* and a male *D. mel*. Rep. indicate replicates 1-3.

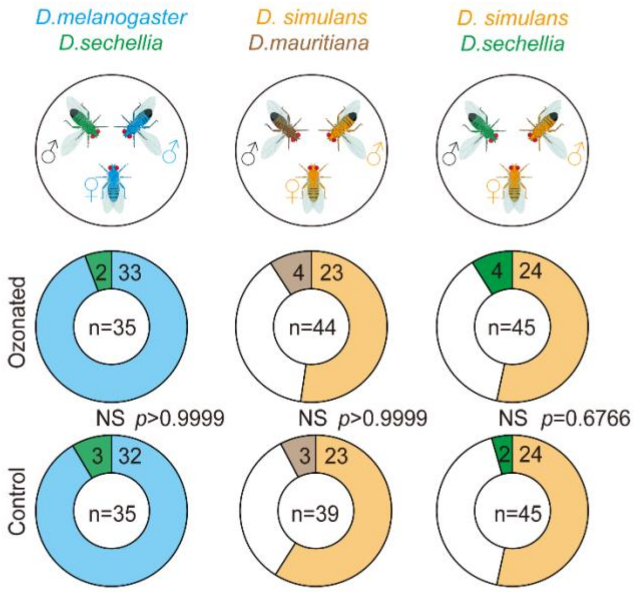

Figure S4. Ozone expose to 50ppb ozone is not enough to induce hybridization among closely related *Drosophila* species. Individual female flies are confronted with one intra- and one interspecific male for six hours. The existence or absence of hybrid offspring informs about the succeeding male. Donut plots of success rates of ozonated (middle) and control (bottom) conspecific and allospecific males courting *D. melanogaster* and *D. simulans*. Sample sizes are provided in donut centers. Numbers in segments depict numbers of successful males. White segments, no male mated the female. Two-tailed *Fisher's exact* test.

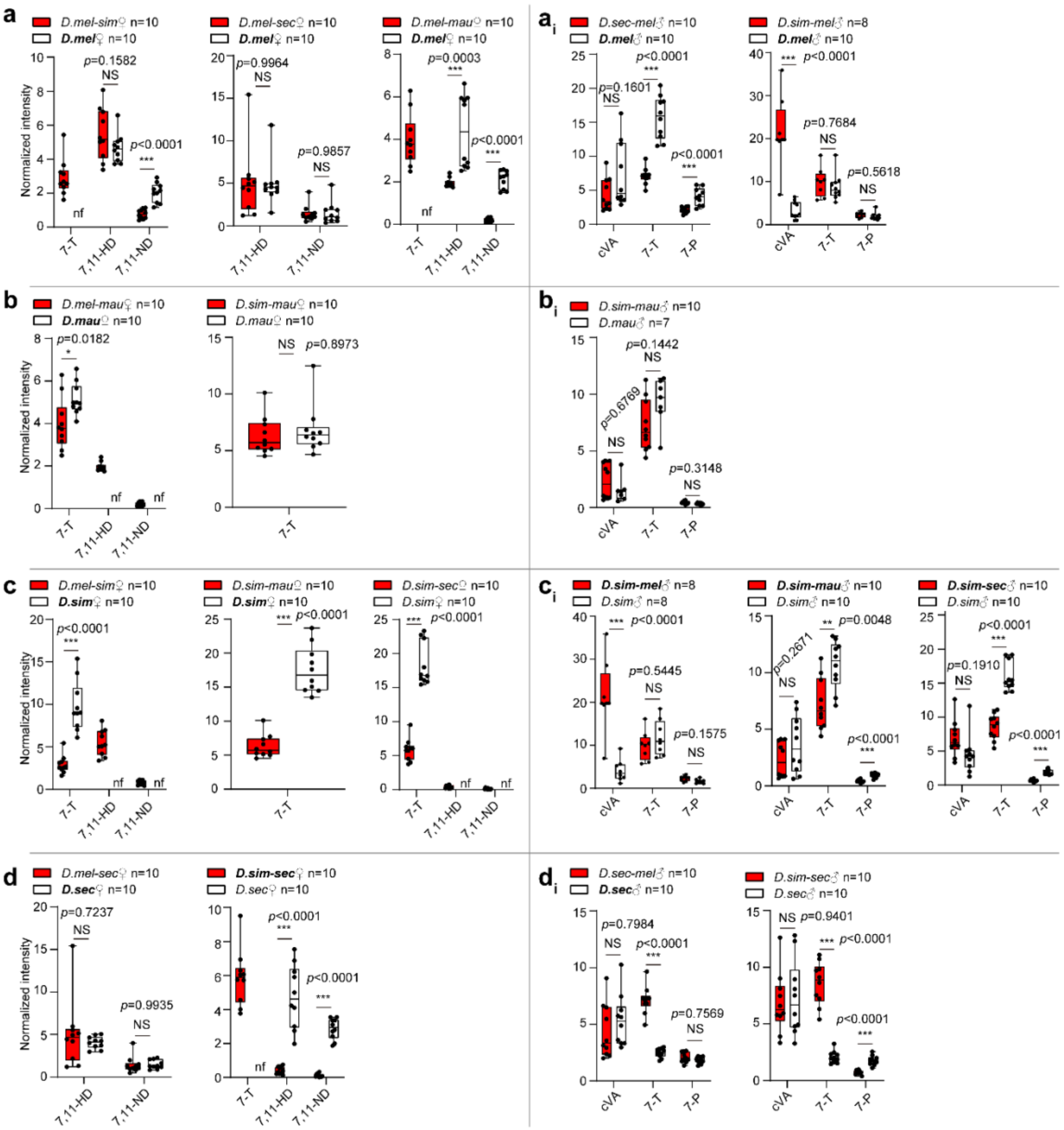

Figure S5. Pheromone quantitative analysis of *D. mel*, *D. sim*, *D. mau*, *D. sec*, and their hybrids. a and **a$_i$**, pheromone of *D. mel* and hybrids. **b** and **b$_i$**, pheromone of *D. mau* and hybrids. **c** and **c$_i$**, pheromone of *D. sim* and hybrids. **d** and **d$_i$**, pheromone of *D. sec* and hybrids. All hybrids are F1 and named as F_0 female × F_0 male. Fly names in bold characters indicate mating preference in competitive mating assays (see Fig.3). The box plots present median values and quartiles, whiskers the minimum and maximum values, and dots the individual data points. *One-way ANOVA* with *Tukey's multiple comparisons* test for hybrid of *D. sim-mau*, *D. mel-sec*, *D. sim-mel*, *D. sec-mel*, and male *D. sim-sec*. While *t*- test for hybrid of *D. mel-mau*, *D. mel-sim*, and female *D. sim-sec*. NS indicate no significant

difference. *$p<0.05$; **$p<0.01$; ***$p<0.001$. While some of the hybrid pheromone patterns correspond well with the observed behavior (e.g. *D. mauritiana* males mate similarly often with *D. sim-mau* females v.s. *D. mauritiana* females, which also share the same pheromone amounts), others do not (e.g. *D. melanogaster* males mate more often with *D. melanogaster* females than with *D. mel-sec* hybrids, although both females share the same pheromones). Obviously other parameters (e.g. the females' acceptance of the male song) play an additional roles here.

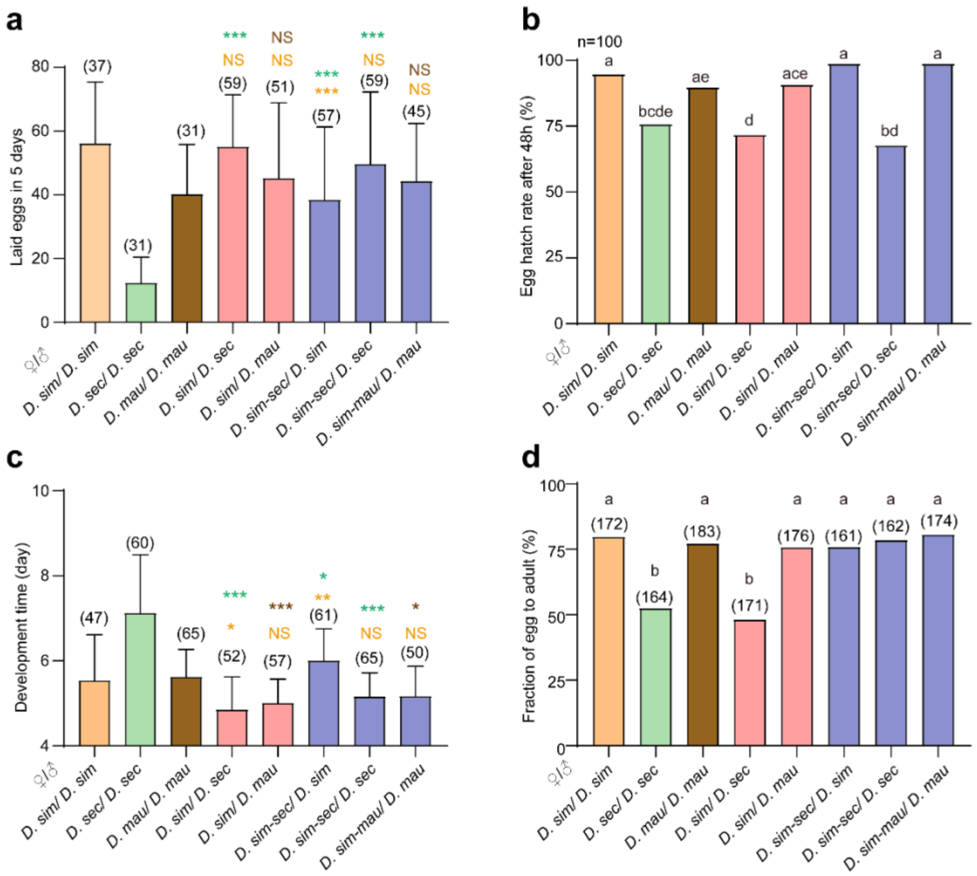

Figure S6. Fitness of female hybrids and purebred flies regarding egg numbers, hatching rates, development time, and the survival rate from egg to adult. a, Egg numbers of each female during 5 days after mating. Figure shows mean ± SD. **b,** Egg hatching rate after 48h. **c**, Development time (days) from egg to pupa. Figure shows mean ± SD. **d**, survival rate from egg to adult. The x-axis shows the parental combination (female/male). *Kruskal Wallis* with *Tukey Kramer post-hoc* test for selected pairs for **a** and **c**. *Chi-square* test with *Bonferroni adjustment* for **b** and **d**. Stars or characters with orange, green, and brown depict the comparison with *D. sim*, *D. sec*. and *D. mau*, respectively. *p<0.05; **p<0.01; ***p<0.001; NS, no significant difference.

Reference:

1 Mallet, J. What does *Drosophila* genetics tell us about speciation? *Trends Ecol Evol* **21**, 386-393, doi:10.1016/j.tree.2006.05.004 (2006).

2 Schliewen, U. K., Tautz, D. & Paabo, S. Sympatric speciation suggested by monophyly of crater lake cichlids. *Nature* **368**, 629-632, doi:10.1038/368629a0 (1994).

3 Shaw, P. W., Turner, G. F., Idid, M. R., Robinson, R. L. & Carvalho, G. R. Genetic population structure indicates sympatric speciation of Lake Malawi pelagic cichlids. *P Roy Soc B-Biol Sci* **267**, 2273-2280, doi:10.1098/rspb.2000.1279 (2000).

4 Meyer, A., Kocher, T. D., Basasibwaki, P. & Wilson, A. C. Monophyletic origin of lake Victoria cichlid fishes suggested by mitochondrial-DNA sequences. *Nature* **347**, 550-553, doi:10.1038/347550a0 (1990).

5 Bush, G. L. Sympatric host race formation and speciation in Frugivorous flies of Genus Rhagoletis (Diptera, Tephritidae). *Evolution* **23**, 237-&, doi:10.2307/2406788 (1969).

6 Feder, J. L., Chilcote, C. A. & Bush, G. L. Genetic differentiation between sympatric host races of the apple maggot fly Rhagoletis pomonella. *Nature* **336**, 61-64, doi:10.1038/336061a0 (1988).

7 Filchak, K. E., Roethele, J. B. & Feder, J. L. Natural selection and sympatric divergence in the apple maggot. *Nature* **407**, 739-742, doi:10.1038/35037578 (2000).

8 Mallet, J. Hybrid speciation. *Nature* **446**, 279-283, doi:10.1038/nature05706 (2007).

9 Mallet, J., Beltrán, M., Neukirchen, W. & Linares, M. Natural hybridization in heliconiine butterflies: the species boundary as a continuum. *Bmc Evol Biol* **7**, doi:10.1186/1471-2148-7-28 (2007).

10 Mavárez, J. *et al.* Speciation by hybridization in butterflies (vol 441, pg 868, 2006). *Nature* **592**, E4-E5, doi:10.1038/s41586-021-03330-8 (2021).

11 Sawamura, K., Sato, H., Lee, C. Y., Kamimura, Y. & Matsuda, M. A natural population derived from species hybridization in the species complex on Penang Island, Malaysia. *Zool Sci* **33**, 467-475, doi:10.2108/zs160038 (2016).

12 Kliman, R. M. *et al.* The population genetics of the origin and divergence of the *Drosophila simulans* complex species. *Genetics* **156**, 1913-1931, doi:10.1093/genetics/156.4.1913 (2000).

13 Coyne, J. A. & Orr, H. A. Patterns of speciation in *Drosophila*. *Evolution* **43**, 362-381 (1989).

14 Turissini, D. A., McGirr, J. A., Patel, S. S., David, J. R. & Matute, D. R. The rate of evolution of postmating-prezygotic reproductive isolation in *Drosophila*. *Mol Biol Evol* **35**, 312-334, doi:10.1093/molbev/msx271 (2018).

15 Coyne, J. A. & Charlesworth, B. Genetics of a pheromonal difference affecting sexual isolation between *Drosophila mauritiana* and *D. sechellia*. *Genetics* **145**, 1015-1030, doi:10.1093/genetics/145.4.1015 (1997).

16 Coyne, J. A. Genetics of differences in pheromonal hydrocarbons between *Drosophila melanogaster* and *D. simulans*. *Genetics* **143**, 353-364, doi:10.1093/genetics/143.1.353 (1996).

17 Coyne, J. A. Genetics of a difference in male cuticular hydrocarbons between two sibling species, *Drosophila simulans* and *D. sechellia*. *Genetics* **143**, 1689-1698, doi:10.1093/genetics/143.4.1689 (1996).

18 Ferveur, J. F. Cuticular hydrocarbons: Their evolution and roles in pheromonal communication. *Behav Genet* **35**, 279-295, doi:10.1007/s10519-005-3220-5 (2005).

19 Cowling, D. E. & Burnet, B. Courtship songs and genetic control of their acoustic characteristics in sibling species of the *Drosophila melanogaster* subgroup. *Anim Behav* **29**, 924-935, doi:10.1016/S0003-3472(81)80030-9 (1981).

20 Tomaru, M. & Oguma, Y. Mate choice in *Drosophila melanogaster* and *D. sechellia*: criteria and their variation depending on courtship song. *Anim Behav* **60**, 797-804, doi:10.1006/anbe.2000.1543 (2000).

21 Tomaru, M., Yamada, H. & Oguma, Y. Female mate recognition and sexual isolation depending on courtship song in and its siblings. *Genes Genet Syst* **79**, 145-150, doi:10.1266/ggs.79.145 (2004).

22 Matute, D. R. & Ayroles, J. F. Hybridization occurs between *Drosophila simulans* and *D. sechellia* in the Seychelles archipelago. *J Evolution Biol* **27**, 1057-1068, doi:10.1111/jeb.12391 (2014).

23 Nunes, M. D. S., Orozco-Ter Wengel, P., Kreissl, M. & Schlotterer, C. Multiple hybridization events between *Drosophila simulans* and *Drosophila mauritiana* are supported by mtDNA introgression. *Mol Ecol* **19**, 4695-4707, doi:10.1111/j.1365-294X.2010.04838.x (2010).

24 Brand, C. L. & Levine, M. T. Cross-species incompatibility between a DNA satellite and the Spartan homolog poisons germline genome integrity. *Curr Biol* **32**, 2962-2971, doi:10.1016/j.cub.2022.05.009 (2022).
25 Barbash, D. A., Awadalla, P. & Tarone, A. M. Functional divergence caused by ancient positive selection of a hybrid incompatibility locus. *Plos Biol* **2**, 839-848, doi:10.1371/journal.pbio.0020142 (2004).
26 Presgraves, D. C. & Meiklejohn, C. D. Hybrid sterility, genetic conflict and complex speciation: Lessons from the clade species. *Frontiers in Genetics* **12**, doi:10.3389/fgene.2021.669045 (2021).
27 Fang, S. *et al.* Incompatibility and competitive exclusion of genomic segments between sibling species. *Plos Genet* **8**, doi:10.1371/journal.pgen.1002795 (2012).
28 Ferree, P. M. & Barbash, D. A. Species-specific heterochromatin prevents mitotic chromosome segregation to cause hybrid lethality in *Drosophila*. *Plos Biol* **7**, doi:10.1371/journal.pbio.1000234 (2009).
29 Coyne, J. A. & Orr, H. A. Patterns of speciation in Drosophila. *Evolution* **43**, 362-381 (1989).
